# Efficient method for transfer of microinjected eggs to mouse ampulla for generating transgenic mice

**DOI:** 10.1186/s40064-016-3760-6

**Published:** 2016-12-05

**Authors:** Guang Wen, Jing Di, Qian Li, Jianling Chen, Ling Jin, Cheng Wang, Sanqing Xu

**Affiliations:** 1 Department of Developmental Neurobiology, New York State Institute for Basic Research in Developmental Disabilities, Staten Island, NY 10314 USA; 2 Department of Biology, College of Staten Island, City University of New York, Staten Island, NY 10314 USA; 3 Department of Neurochemistry, New York State Institute for Basic Research in Developmental Disabilities, Staten Island, NY 10314 USA; 4 Department of Pediatrics, Tongji Hospital, Huazhong University of Science and Technology, Wuhan, 430030 Hubei China

**Keywords:** New device, Microinjection of eggs, Transgenic/knockout mice, Generation of transgenic/knockdown mice

## Abstract

**Background:**

The new method described here is highly efficient in transferring microinjected mouse eggs (MEs) through the bursa membrane of a surrogate mother mouse to the ampulla of the oviduct without damaging the blood vessels on the bursa membrane.

**Results:**

This method causes no loss of blood, and it produces newborn pups/founders from approximately 70% of the transferred MEs, because only a small hole is made on the blood vessel–free area of the bursa membrane and ampulla of the surrogate mother mouse. The infundibulum remains intact. The small hole on the bursa membrane/ampulla may already heal up before the delivery of the new pups. The method described here consists of a simple operation with a home-assembled drill head holding a self-closing fine forceps on one end, while the drill head assembly body is hooked up with the light housing clamp of a dissecting light microscope. The drill head assembly body can be alternatively hooked/tied up to an appropriate size of clamp (purchased from Home Depot) screwed to any light stand with folding segments.

**Conclusion:**

This system is able to steadily hold the self-closing fine forceps without shaking and to let the operator use their two hands to steadily hold and quickly insert the pipet carrying the MEs into the ampulla without any delay. Generally MEs stay alive for approximately 15 min at room temperature. The shorter the insertion time is, the more MEs that will survive. Thus, this method may produces more pups/founders.

**Electronic supplementary material:**

The online version of this article (doi:10.1186/s40064-016-3760-6) contains supplementary material, which is available to authorized users.

## Background

In the current method for producing transgenic/knockdown mice, the bursa membrane of a surrogate mother mouse is generally opened or torn apart to expose the infundibulum before a pipet tip containing microinjected mouse eggs (MEs; eggs also referred to as embryos) is transferred/inserted through the top opening of the infundibulum to the ampulla of the surrogate mouse (Behringer et al. 2014; Wen and Chen 2004; Hogan et al. 1994). This task is rather challenging, because blood from the blood vessels of the bursa membrane floods and covers up the infundibulum opening and ampulla. As a result, it eventually becomes impossible for the operator to see the top opening of the infundibulum/ampulla before inserting the pipet tip to transfer the MEs (Behringer et al. 2014; Wen and Chen 2004). Because the bursa membrane and oviduct are slippery, it is difficult to punch a small hole through them with a 29-gauge needle or any small needle without holding the bursa membrane and infundibulum together with a fine, self-closing forceps (Liu et al. 2013), because the bursa membrane and ampulla will move around. The new method described here overcomes these two problems. In addition, because our device holds the bursa membrane and infundibulum stably with self-closing forceps, it frees the investigator’s two hands to perform a quick transfer/injection of the MEs to the ampulla. The shorter the transfer/time, the higher the survival rate of the MEs at room temperature. It is therefore easy to achieve newborn pups/founders from 70% of these MEs.

## Methods

### Animals

Mice of CD-1 strain (*Mus musculus*) were purchased from the Charles River Laboratory (Wilmington, MA). Mice were cared for and maintained humanely in our animal colony, under proper housing and husbandry conditions conforming to the U.S. Public Health Service guidelines for humane care and use of laboratory animals of the National Research Council (2011) and the National Institutes of Health (1985). All of the new and used cages, air filters, water feeding bottles, and other items used to care for these animals were cleaned and autoclaved, as a pathogen-free environment is required for every animal in our animal colony. In addition, this study was part of our project entitled “Production and Breeding of Neuroligin-3 and -4 Knock Down Mice for Autism Research,” which was approved by the Animal Welfare Committee of our Institute (PR#330).

The mice were serologically tested by Charles River Laboratory to confirm the absence of the following pathogens: Sendai virus, pneumonia virus of mice, mouse hepatitis virus, minute virus of mice, Theiler’s virus, reovirus, *Mycoplasma pulmonis*, lymphocytic choriomeningitis virus, ectromelia virus, mouse pneumonitis virus, polyoma virus, mouse adenovirus FL/K87 1 & 2, epizootic diarrhea of infant mice virus, mouse cytomegalovirus, Hantaan virus, *Encephaokitozoon cuniculi*, cilia-associated respiratory bacillus, mouse parvovirus, and mouse thymic virus. Mice were housed in a quarantine facility at our animal colony with a 12-h light–12-h dark cycle and 10–15 air changes/h. Mice were housed on sterilized corncob bedding (Harlan, Indianapolis, IN) in sterilized cages (polysaufone Standard mouse cage, ACE, Allentown, PA) equipped with stainless steel wire bar tops and filtered cage tops. Mice were fed with Lab Diet 5015, Mouse Diet (PMI Nutrition International, Inc., Brentwood, MO) and hyperchlorinated water available ad libitum in bottles. Cage litter was changed every seven days in a class-II biological safety cabinet in the animal colony.

**Fig. 1:**
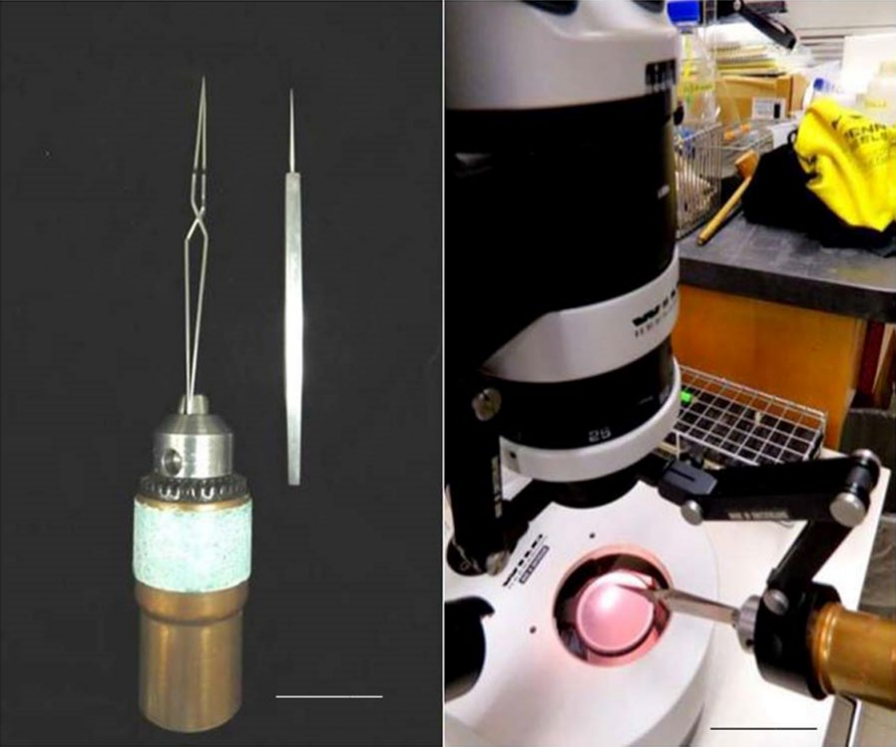
Microinjected egg transfer assembly. *Left panel* Drill head assembly holding a fine forceps (*left*) and a fine needle (*right*) [*scale bar* 20 mm]. *Right panel* The drill head assembly with the fine forceps held in the holder of the light housing clamp of a dissecting light microscope (*Scale bar* 30 mm)

**Fig. 2:**
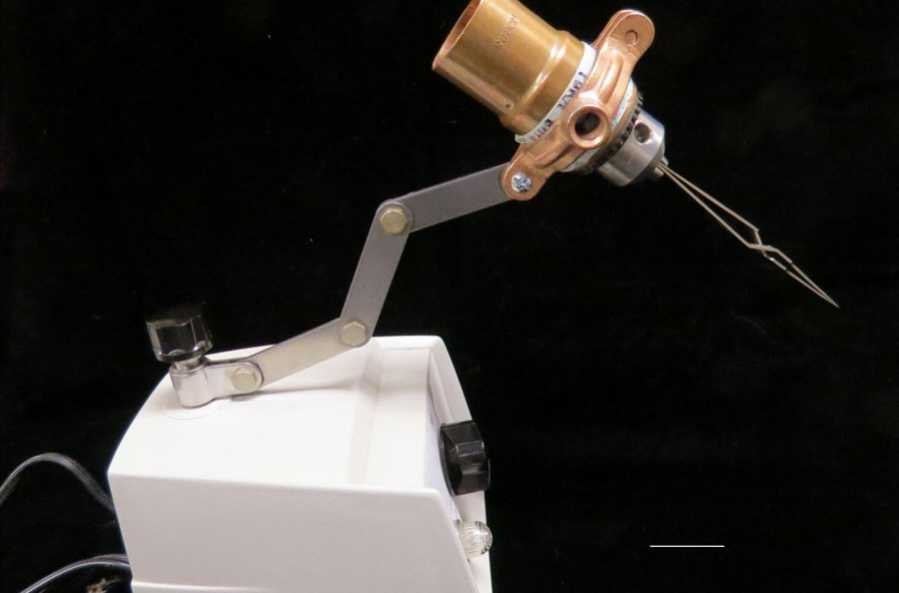
Drill head assembly holding a self-closing fine forceps can be alternatively hooked/tied up to an appropriate size of clamp screwed to any light stand with folding segments (*Scale bar* 30 mm)

Superovulation in female mice around 28 days old was achieved by intraperitoneal (i.p.) administration of 5–7.5 IU (=0.1–0.15 ml of 50 IU/ml) of pregnant mare’s serum/ml (Sigma-Aldrich, St. Louis, MO) 47 h prior to i.p. administration of 5–7.5 IU (=0.1–0.15 ml of 50 IU/ml) of human chorionic gonadotropin/ml (Sigma-Aldrich, St. Louis, MO). Each female mouse was then placed in a cage with a fertile stud male mouse at around 4–5 p.m.; the male and female mice were left together overnight. The presence of a copulation plug in the vagina the next morning generally indicated that the female mouse was carrying fertilized eggs/oocytes. All mice were sacrificed by i.p. administration of an overdose (>0.017 ml/body weight in grams) of 2.5% Avertin anesthetic (Sigma-Aldrich). Fertilized eggs (i.e., the presence of two pronuclei in each egg) were selected and microinjected with the desired gene construct under an inverted light microscope equipped with a microinjector and micromanipulator in the standard fashion. Without the bursa membrane being torn apart, the MEs were inserted directly through a small hole made on an area of the bursa membrane without any blood vessels and the ampulla wall to the ampulla cavity by using the following egg-transferring assembly (Figs. 1, 2).

### Instruments needed to set up application of the new egg-transferring assembly (Fig. 1)

1. Self-closing fine forceps (#N5 INOX from A. Dumont & Fils, Switzerland). 2. Fine needle (Miltex, Germany), sharpened with electric grinder and further smoothed out with a fine grinding stone. 3. Drill head assembly (purchased from Home Depot). 4. Pipet tip for holding microinjected eggs.

### Procedures to set up egg-transferal assembly (Fig. 1) and steps for transferring microinjected eggs (MEs)

1. Put the dull end of a self-closing fine forceps into the holding end/cavity of a drill head assembly, and turn tightly to hold the forceps while its two tips are still closed (touching each other) (Fig. 1, left panel).

2. Place the base of the drill head assembly holding the self-closing fine forceps into the holder of the light clamp of the dissecting light microscope (Fig. 1, right panel).

Note: The drill head assembly body can be alternatively hooked/tied up to an appropriate size of clamp (purchased from Home Depot) screwed to any light stand with folding segments (Fig. 2).

**Fig. 3:**
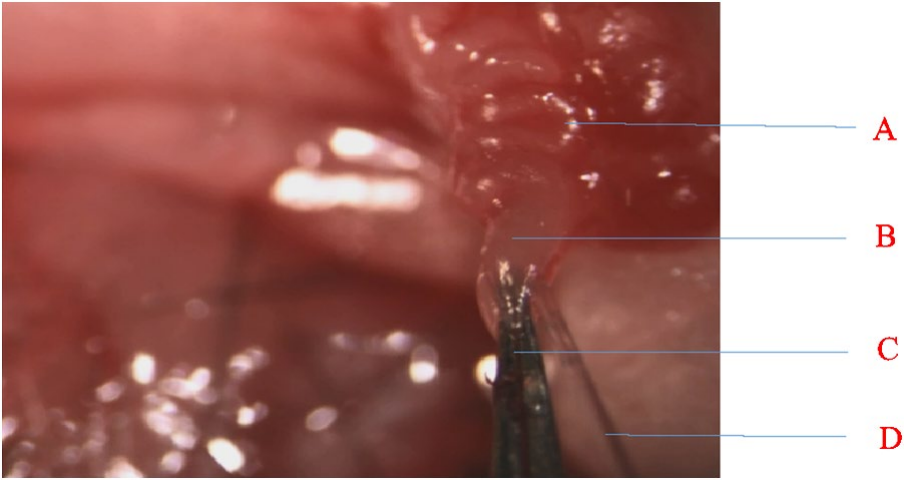
This video-clip link demonstrates that there is no breeding problem when the bursa membrane/ampulla was inserted with pipet tip containing microinjected eggs. A = oviduct; B = ampulla/bursa membrane; C = fine forceps; D = pipet tip containing MEs

**Fig. 4:**
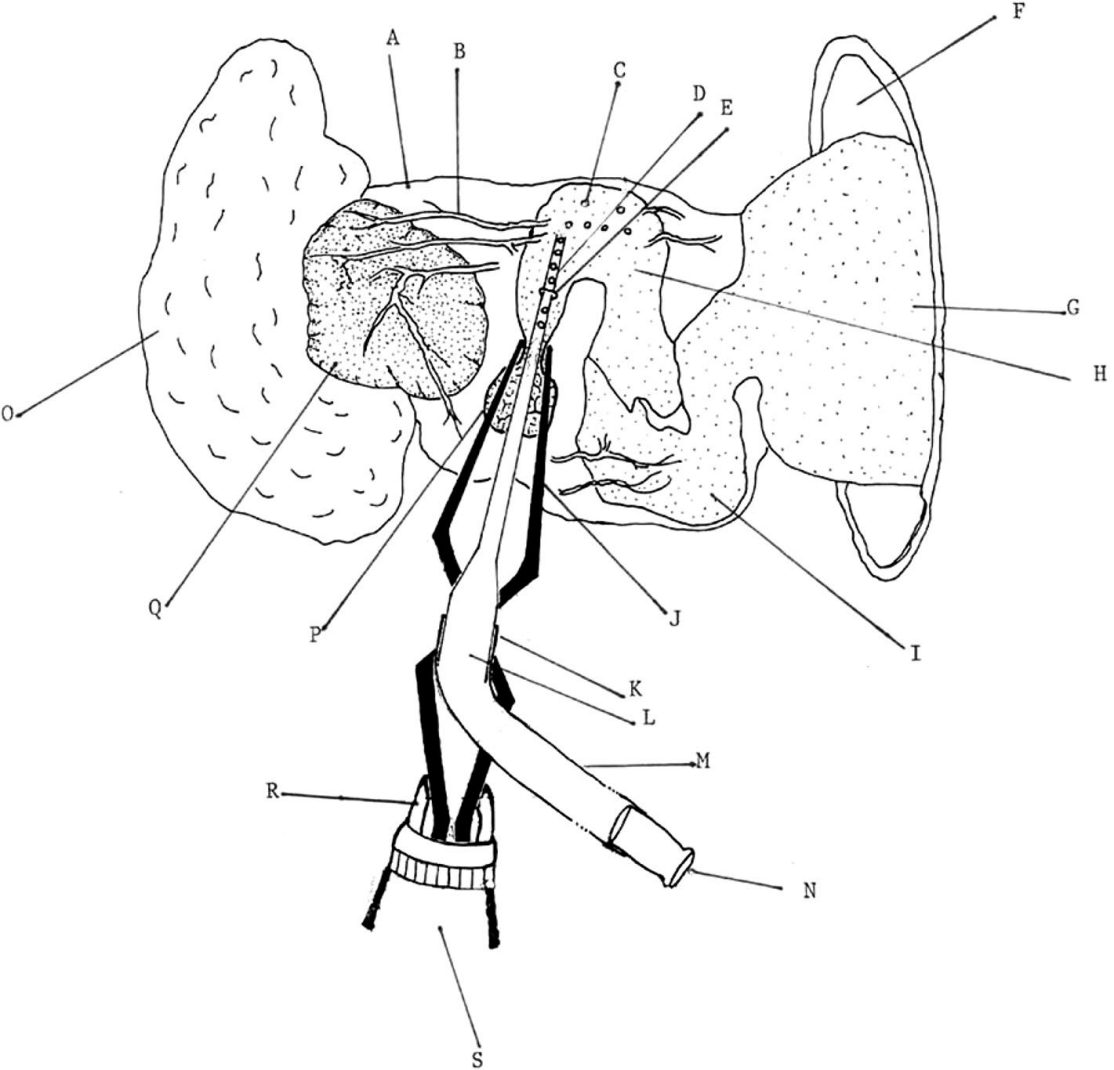
Diagram of a new device and approach to transfer microinjected eggs to mouse ampulla for increased production of transgenic mice. A = bursa membrane; B = blood vessel of bursa membrane; C = microinjected egg; D = pipet tip; E = small hole made for inserting pipet tip; F = surgical hole in mouse body for pulling out ovary, oviduct, and uterus; G = uterus; H = ampulla of oviduct; I = oviduct; J = fine forceps; K = rubber tube attached to pipet; L = pipet end for attaching rubber tube of mouthpiece; M = rubber tube attached to mouthpiece; N = mouth piece; O = fat pad; P = top of infundibulum; Q = ovary; R = head of drill to hold fine forceps; S = drill head assembly

3. Bring the tip of the self-closing fine forceps to the focus point under a dissecting light microscope.

4. Anesthetize the surrogate mother mouse, and surgically open the dorsal midline and move the skin down to abdomen area showing pinkish/red color indicating that it is the location of ovary and uterus. Then, the ovary is surgically exposed through an incision in order to take out the ovary, oviduct, and uterus from the body cavity to prepare the mouse for receiving the MEs.

5. Wet a piece of cheese cloth with sterilized saline, and with it, cover the surface of the ovary and oviduct to keep these organs wet until 10–15 MEs are loaded to the tip of a glass pipet (inner diameter: 180 μm; outer diameter: 200 μm) (Figs. 3, 4D, and a movie-clip as shown in Additional file 1) with a rubber tube (Fig. 4K, M) attached to the other end of this pipet (Fig. 4L).

6. Open the two tips of the self-closing fine forceps (Fig. 1, left panel) to hold the infundibulum and ampulla of the surrogate mother mouse.

7. Remove the cheese cloth, and use a sterilized fine needle (300 μm diameter, the same size as a 29-gauge hypodermic needle) to make a small hole (Fig. 4E) on an area of the bursa membrane without any blood vessels to further penetrate the wall of the ampulla.

8. Insert the pipet tip containing 10–15 MEs through this newly made small hole (Fig. 4E), and blow all MEs including the air bubbles into the internal cavity of the ampulla. The presence of an air bubble inside the cavity of the ampulla indicates that all of the MEs have entered the ampulla cavity.

9. Quickly withdraw the pipet tip, and push the ovary, oviduct, and uterus back into the body cavity of the surrogate mother mouse, and clamp the skin with mechanical staples to seal the wound.

10. Finally, wrap the mouse in a piece of absorbent pad with a plastic lining on one side and an absorbent surface on the other side. Make sure to place the plastic lining next to the mouse’s body to keep it warm without losing heat.

11. Return the animals to the animal colony for daily care management until the birth of the new pups.

### New method for transferring eggs to ampulla

With a 29-gauge needle or a homemade stainless needle (Fig. 1, left panel), we made a small hole through the bursa membrane and ampulla while the closed end of self-closing fine forceps was held by a drill head, as shown in Fig. 1 (left and right panels). Under a dissecting light microscope, a pipet tip loaded with 13 MEs was inserted through the small hole made on both the bursa membrane and the ampulla to the ampulla cavity, and we blew out all of the MEs, plus one air bubble behind the last egg, into the ampulla cavity of one surrogate mouse. In addition, three groups of 14, 8, and 7 MEs were separately inserted into each one of three other surrogate mother mice.

### Old method for transferring eggs to infundibulum/ampulla

In the traditional method, the bursa membrane is torn to expose the opening end of the infundibulum before the tip of a pipet containing MEs is inserted either into this opening or directly into the ampulla cavity. Next, all of the MEs, plus one air bubble behind the last egg, are blown into the ampulla cavity of the surrogate mouse. The blood from the blood vessels of the torn bursa membrane quickly floods and covers up the infundibulum opening and ampulla. The operator needs to quickly complete the insertion of these MEs into the infundibulum opening/ampulla, which is a difficult task.

## Results

The first group of 13 MEs, the second group of 14 MEs, the third group of 8 MEs, and the fourth group of 7 MEs were inserted separately into the ampulla cavities of four different surrogate mother mice. These four surrogate mice delivered 9, 9, 6, and 5 new pups, respectively. Based on these four cases, the success rates of delivering new pups from MEs were in the range of 64–75% (Table 1).

**Table 1:** Numbers of pups delivered by surrogate mother mice that received microinjected eggs with traditional and new transfer methods

Traditional method			New method		
Pups born	Numbers of eggs transferred	% of pups born	Pups born	Numbers of eggs transferred	% of pups born
2	7	28.5%	6	8	75%
1	10	10	9	13	70
1	8	12.5	9	14	64
5	9	55	5	7	71.4
Total 9	34	26.5%	29	42	69.0%

Wilcoxon-Mann–Whitney-test analysis was performed. The z = − 2.309 and p = 0.0209 indicate that the difference between the numbers of pups born in the traditional and the new transferal method is significant

## Discussion and conclusion

With the use of the new protocols described above, no blood was lost from the insertion area of the bursa membrane/ampulla because the 29-gauge needle or other small needle was inserted specifically into an area without any blood vessels in the bursa membrane. No blood vessels were damaged, and no bleeding problems occurred (Fig. 3; Additional file 1). Thus, this protocol makes it possible to visualize the ampulla in order to make a small hole for transferring the MEs, and it achieves high efficiency (64–70%) in producing new pups.

The method described here consists of a simple operation with a home-assembled drill head holding a self-closing fine forceps on one end, while the drill head assembly body is hooked up with the light-housing clamp of a dissecting light microscope. Thus, this system provides a steady grasp of the self-closing fine forceps without shaking and lets operators use their two hands to steadily hold and quickly insert the pipet carrying the MEs into the ampulla without any delay. The shorter the insertion time, the higher the survival rate of the MEs and thus the higher the number of new pups/founders produced. Alternatively, the drill head holding a self-closing fine forceps can be hooked/tied up to an appropriate size of clamp (purchased from Home Depot) screwed to any light stand with folding segments (Fig. 2).

## Additional file

**Additional file 1.** Short video-clip illustrating the entire motion steps from insertion of a pipet tip containing microinjected eggs to a small hole on bursa membrane/ampulla held by a self-closing fine forceps until the completion of transfer action (To open video-clip, mouse left click twice and then open).

## Abbreviations

MEs: mouse eggs
IU: international unit
