# Supplementary material for: Efficient method for transfer of microinjected eggs to mouse ampulla for generating transgenic mice
Source: Springerplus. 2016 Dec 5;5(1):2076. doi: 10.1186/s40064-016-3760-6 (PMC5138171; doi:10.1186/s40064-016-3760-6)
Supplement: Supplementary file 1 — Additional file 1. Short video-clip illustrating the entire motion steps from insertion of a pipet tip containing microinjected eggs to a small hole on bursa membrane/ampulla held by a self-closing fine forceps until the completion of transfer action (To open video-clip, mouse left click twice and then open). [file 40064_2016_3760_MOESM1_ESM.docx]

Additional Data File /Movie file (To open this additional data file, mouse left click

twice and then open).
